# Supplementary material for: Mechanisms governing the pioneering and redistribution capabilities of the non-classical pioneer PU.1
Source: Nat Commun. 2020 Jan 21;11:402. doi: 10.1038/s41467-019-13960-2 (PMC6972792; doi:10.1038/s41467-019-13960-2)
Supplement: Supplementary file 7 — Source data [file 41467_2019_13960_MOESM7_ESM.zip › Source_Data/Figure6/Figure6B_MotifScanOutput/FLI1_mutPU.1/homerResults/motif22.similar.html]

motif22

## Information for motif22

A
G
C
T
A
C
G
T
A
C
G
T
A
G
T
C
A
G
C
T
C
T
G
A
C
T
G
A
A
C
T
G
C
G
T
A
C
G
T
A
  
Reverse Opposite:  

A
C
G
T
A
C
G
T
A
G
T
C
A
G
C
T
G
A
C
T
C
T
G
A
A
C
T
G
C
G
T
A
C
G
T
A
T
C
G
A
  

|  |  |
| --- | --- |
| p-value: | 1e-47 |
| log p-value: | -1.095e+02 |
| Information Content per bp: | 1.842 |
| Number of Target Sequences with motif | 523.0 |
| Percentage of Target Sequences with motif | 5.10% |
| Number of Background Sequences with motif | 844.7 |
| Percentage of Background Sequences with motif | 2.22% |
| Average Position of motif in Targets | 102.3 +/- 54.0bp |
| Average Position of motif in Background | 100.9 +/- 65.0bp |
| Strand Bias (log2 ratio + to - strand density) | -0.1 |
| Multiplicity (# of sites on avg that occur together) | 1.06 |
| Motif File: | file (matrix) reverse opposite |

### Similar de novo motifs found

|  |  |  |  |  |  |  |  |
| --- | --- | --- | --- | --- | --- | --- | --- |
| Rank | Match Score | Redundant Motif | P-value | log P-value | % of Targets | % of Background | Motif file |
| 1 | 0.969 | A T C G T C G A A G C T A C G T A C G T A G T C A G C T C G T A C T G A A C T G C G T A C G T A C T G A | 1e-43 | -101.136128 | 3.52% | 1.29% | motif file (matrix) |
| 2 | 0.861 | A G C T C G A T T A G C A T C G G T A C T C G A A T C G G T C A C T G A | 1e-36 | -84.699835 | 4.54% | 2.11% | motif file (matrix) |
| 3 | 0.962 | T A G C C T A G G C A T A G C T C G A T A T G C G A C T A T G C C G T A C T A G T G C A G T C A | 1e-36 | -83.720227 | 3.51% | 1.44% | motif file (matrix) |
| 4 | 0.941 | A G C T A G C T C G A T A T G C A G C T G A T C C T G A C T A G C G T A C G T A A C G T | 1e-33 | -77.802029 | 2.87% | 1.09% | motif file (matrix) |
